# 4-[^18^F]Fluorophenylpiperazines by Improved Hartwig-Buchwald *N*-Arylation of 4-[^18^F]fluoroiodobenzene, Formed via Hypervalent λ^3^-Iodane Precursors: Application to Build-Up of the Dopamine D_4_ Ligand [^18^F]FAUC 316

**DOI:** 10.3390/molecules20010470

**Published:** 2014-12-31

**Authors:** Fabian Kügler, Johannes Ermert, Peter Kaufholz, Heinz H. Coenen

**Affiliations:** Institute of Neuroscience and Medicine, INM-5: Nuclear Chemistry, Forschungszentrum Jülich, 52425 Jülich, Germany; E-Mails: fabian.kuegler@petnet-solutions.de (F.K.); p.kaufholz@fz-juelich.de (P.K.); h.h.coenen@fz-juelich.de (H.H.C.)

**Keywords:** fluorine-18, Hartwig-Buchwald *N*-arylation, dopamine D_4_ radioligand, iodophenyl-iodonium compounds, iodonium ylides

## Abstract

Substituted phenylpiperazines are often neuropharmacologically active compounds and in many cases are essential pharmacophores of neuroligands for different receptors such as D_2_-like dopaminergic, serotoninergic and other receptors. Nucleophilic, no-carrier-added (n.c.a.) ^18^F-labelling of these ligands in an aromatic position is desirable for studying receptors with *in vivo* molecular imaging. 1-(4-[^18^F]Fluorophenyl)piperazine was synthesized in two reaction steps starting by ^18^F-labelling of a iodobenzene-iodonium precursor, followed by Pd-catalyzed *N*-arylation of the intermediate 4-[^18^F]fluoro-iodobenzene. Different palladium catalysts and solvents were tested with particular attention to the polar solvents dimethylformamide (DMF) and dimethylsulfoxide (DMSO). Weak inorganic bases like potassium phosphate or cesium carbonate seem to be essential for the arylation step and lead to conversation rates above 70% in DMF which is comparable to those in typically used toluene. In DMSO even quantitative conversation was observed. Overall radiochemical yields of up to 40% and 60% in DMF and DMSO, respectively, were reached depending on the labelling yield of the first step. The fluorophenylpiperazine obtained was coupled in a third reaction step with 2-formyl-1*H*-indole-5-carbonitrile to yield the highly selective dopamine D_4_ ligand [^18^F]FAUC 316.

## 1. Introduction

Since their first uses in the1960s, cross-coupling reactions using Pd(0) complexes play a major role in organic syntheses to form carbon-carbon or carbon-heteroatom bonds [1]. This was recently also recognized by awarding the Nobel Prize to Richard F. Heck, Ei-ichi Negishi, and Akira Suzuki in 2010 [2]. Therefore, it is remarkable that application of this method in radiosyntheses using short-lived radionuclides like ^11^C (t_1/2_ = 20.4 min) or ^18^F (t_1/2_ = 109.7 min) is still an exception and only a few examples of its use are described [3,4,5]. Cross-coupling reactions allow for a quick synthesis of many structure motives of biomolecules relevant for non-invasive diagnostic imaging techniques such as positron emission tomography (PET). Reasons for the rare use of palladium-mediated coupling methods in radiochemistry with positron-emitters may be the following:

- Whenever possible, multi-step reactions are avoided in radiosyntheses with short-lived nuclides.
- Syntheses with many reactants often lead to problems of reproducibility and optimization under no-carrier-added (n.c.a.) conditions and complicate a rapid on-line separation of products.
- The typical use of non-polar solvents and dry reaction conditions for the coupling reaction necessitate an extensive pre-separation of labelled intermediates which is often time and yield consuming.
- Insoluble compounds (mixture of organic and inorganic reactants) complicate the applicability of the method in remotely controlled synthesis devices.

Otherwise none of these problems are insuperable, and new technical developments, like microfluidic systems, may help to solve the problem with solubility [6,7]. Since diaryliodonium salts allow to access n.c.a. [^18^F]fluorohalobenzenes in high yields and in one reaction step [8,9,10] the availability of ^18^F-labelled electrophilic coupling partners is not any longer a hindrance to apply such coupling methods [5,11,12,13,14].

One basic cross-coupling reaction is the Hartwig-Buchwald *N*-arylation (HBC), which leads to arylamines, important structure elements of a lot of bioactive compounds. Although alternative pathways for generating fluoroarylamines have been used including direct fluorination, these proved not to be practical for n.c.a. ^18^F-labelled arylamine compounds. *Para*-alkylamine substituents such as piperidine or piperazine cause a maximum slow-down of reaction kinetics of S_N_Ar reactions, what is reflected by very low Hammett σ constants of about −1 of those substituents [15]. Direct labelling of arylamines with [^18^F]fluoride was therefore impossible so far. Alternatively, three-step syntheses of n.c.a. [^18^F]fluorophenylpiperazine have been developed starting from dinitrobenzene derivatives [16,17].

Thus, the HBC offers the possibility of a two-step procedure to ^18^F-labelled arylpiperazine or piperidines. The choice of the solvent is the main challenge is of this reaction. Dimethylformamide (DMF) which is commonly used for ^18^F-labelling of iodonium salts failed as solvent for HBC in previous studies [18,19]. The aim of this work, therefore, was the optimization of HB-coupling conditions with n.c.a. ^18^F-labelled iodobenzene to allow for the use of polar media with regard to facilitate a one-pot synthesis of 1-(4-[^18^F]fluorophenyl)piperazine. Such reaction conditions avoid both pre-separation and pre-drying, and may result in a definite increase of efficiency of HBC in radiochemistry.

1-(4-Fluorophenyl)piperazine is itself a neuropharmacologically active substance [20] and thus interesting as an imaging probe, but it was not ^18^F-labelled so far. The actual benefit of this molecular structure for the development of pharmaceuticals lies in the possibility of easy-to-perform coupling by reductive amination (mild conditions, toleration of moisture and of many functional groups, high yields and reproducibility) of different aldehyde moieties in order to synthesize various phenylbenzyl-piperazines. These are important structure motives for different neuroligands, including ligands for D_2_-like dopamine receptors, serotonin receptors, sigma receptors [21], adrenergic receptors [22] and calcium channels [23]. In many cases, an aromatic fluorine substituent increases the affinity and/or selectivity of a ligand, which is important for its use in radiodiagnostics [24,25].

**Figure 1:**
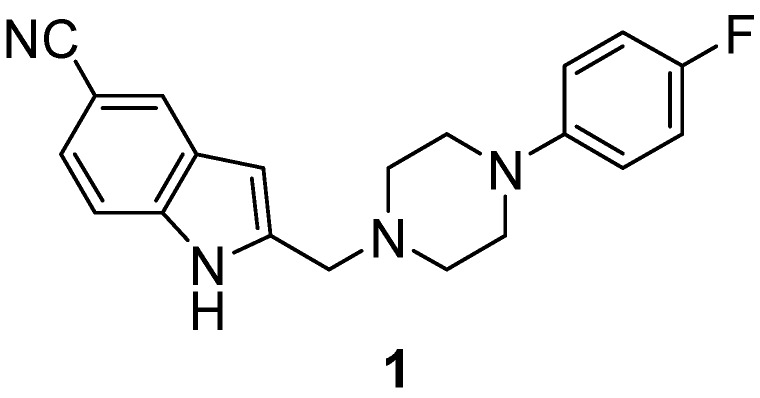
FAUC 316, one of the most selective ligands known for the D_4 _ receptor.

One very interesting ligand of this group is the highly affine (K_i_(D_4_) = 1 nM) and selective (K_i_(D_1-3_) = 8600–28,000 nM) dopamine D_4_ ligand FAUC 316 (**1**) (see Figure 1) [26]. A direct radiolabelling of this compound by fluorine-18 or carbon-11 was not carried out so far. However, in order to achieve an efficient radiolabelling of FAUC analogues, two fluoroethoxy-substituted derivatives, namely 2-[4-(2-(2-fluoro-ethoxy)phenyl)-piperazin-1-ylmethyl]indole-5-carbonitrile and 2-[4-(4-(2-fluoroethoxy)-phenyl)-piperazin-1-ylmethyl]indole-5-carbonitrile, have been developed which were ^18^F-labelled with radiochemical yields of 80% and 47%, respectively [27]. Their respective Ki values of 2.1 and 9.9 nM for the dopamine D_4_ receptor subtype are comparable with the Ki value of 1 nM of FAUC 316. However, the D_4_ selectivity within the D_2_ family, best displayed by a 420-fold D_4_-selectivity over D_2_ receptors by 2-[4-(4-(2-fluoroethoxy)-phenyl)piperazin-1-ylmethyl]indole-5-carbonitrile, is much lower compared to the factor of 19,000 for FAUC 316. Furthermore, a new series of FAUC derivatives, derived from aminomethyl-substituted pyrazolo[1,5-a]pyridine (FAUC 113 and 213), have been pharmacologically evaluated and labelled with fluorine-18 [28]. The best derivative of this series, labelled by ^18^F-fluoroethylation with 88% RCY, showed a Ki value of 13 nM which again is by a factor of about 10 lower than that of FAUC 316. The dopamine D_4_ receptor exhibits a real challenge with regard to its* in vivo* determination due to the extremely low concentration (B_max_(brain) ≈ 9–30 fmol/g(tissue)) of this receptor subtype in the mammalian brain [29]. Therefore, a suitable radioligand for PET or an alternative molecular imaging method does not exist until now [30,31].

Due to the strong and multiple effects of dopamine on the human body it is difficult to differentiate between the individual influences of the 5 dopamine subtypes since they show a very similar binding behavior [32,33]. This similarity poses a high demand on the selectivity of ligands for imaging of an individual subtype of dopaminergic receptors.

## 2. Results and Discussion

### 2.1. Palladium-Mediated N-Arylation

The synthesis of an n.c.a. 4-[^18^F]fluorohalobenzene as electrophilic coupling compound for HBC was conducted by using hypervalent λ^3^-iodane (also called iodonium) species as ^18^F-labelling precursor. Although the exact labelling mechanism of the nucleophilic substitution on iodo(III) arene compounds with fluoride is not known until now [34], it lead to [^18^F]fluoroaryls also with poorly activating and even electron withdrawing substituents in high yields. Ideally, it is desirable that all [^18^F]fluoroarenes are synthesized by direct labelling of, e.g., a corresponding iodonium compound. In reality, however, the synthesis of those precursors is limited since it requires harsh oxidative conditions with a rather poor tolerance of functional groups, and extremely low labelling yields are often obtained from more complex iodonium precursors. Especially nitrogen-containing aryliodonium salts are difficult to synthesize by classical routes for diaryliodonium salts [35]. New modifications were developed to overcome this challenge [36,37], but only very few examples of complex nitrogen-containing iodonium salts can be found in the literature [38,39,40]. The fact that all of the complex iodonium precursors used for a successful ^18^F-labelling do not contain an amine group, is only an empirically based information. However, it was verified in our study with the example of 4-(4-benzylpiperazin-1-yl)phenyl(4-methoxyphenyl)iodonium triflate. No ^18^F-labelling could be observed using this precursor; that is why a direct one-step synthesis of [^18^F]FAUC 316 ([^18^F]**1**) failed. Therefore, multi-step reactions are still necessary for ^18^F-fluorination of these electron rich aryls, where hypervalent λ^3^-iodane species, nevertheless, serve as excellent starting material.

**Figure 2:**
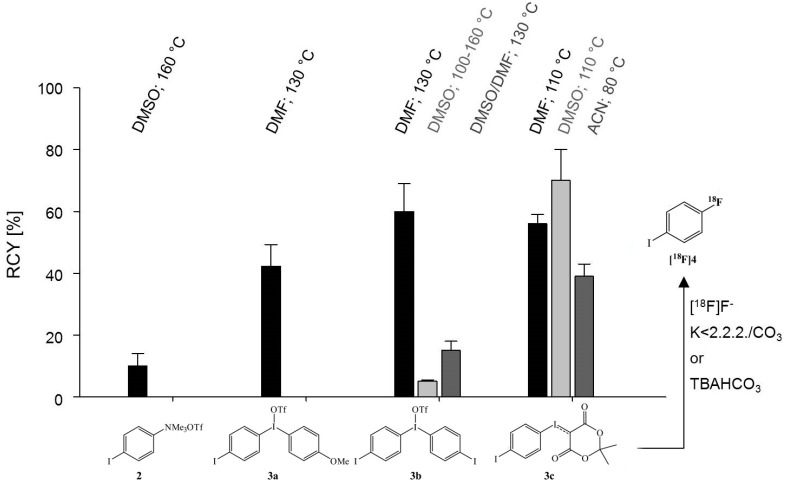
Radiosynthesis of 1-[^18^F]fluoro-4-iodobenzene ([^18^F]**4**) by nucleophilic substitution on iodo(III)arenes under various reaction conditions (*n* = 3–6).

In this work, 1-[^18^F]fluoro-4-iodobenzene ([^18^F]**4**) was used rather than the bromine-analogue because higher labelling yields were found and preliminary examinations showed even slightly higher coupling rates. For comparison, an ^18^F-for-N(CH_3_)_3_ exchange on 4-iodophenyltrimethylammonium triflate (**2**) yielded only 12% ± 3% in DMSO at 160 °C, while the unsymmetrical iodonium salt, 4-iodophenyl-(4′-methoxyphenyl)-iodonium triflate (**3a**), yielded [^18^F]**4** at 130 °C in DMF with 40% ± 7% (Figure 2). Since a convenient synthesis of the symmetrical bis(4-iodophenyl)iodonium salt (**3b**) was accessible [41], highest labelling yields of 60% ± 9% could be reached with this iodonium precursor under the same conditions using the Kryptofix^®^2.2.2./potassium carbonate system, without any additive or scavenger which was recommended by Carroll* et al.* [42]. The solvents DMSO and mixtures of DMSO/DMF proved less suitable for the ^18^F-fluorination of the corresponding diaryliodonium salts.

A novel precursor, however, (2,2-dimethyl-5,7-dioxo-1,3-dioxocan-6-yl)(4-iodophenyl)iodonium ylide (**3c**) [43], gave still a higher RCY of 70% ± 10%. In contrast to the other precursors the best RCY was again obtained in dimethylsulfoxide (DMSO) and tetrabutylammonium bicarbonate as anion activator. Furthermore, a lower reaction temperature of 110 °C could be employed. It is notable, that even in acetonitrile a RCY up to 38% of [^18^F]**4** was obtained when using the iodonium ylide.

During the preparation of this manuscript, however, Mu* et al.* published an alternative method for the synthesis of 1-[^18^F]fluoro-4-iodobenzene ([^18^F]**4**) using triarylsulfonium salts which lead even to 90% RCY of n.c.a. 1-[^18^F]fluoro-4-iodobenzene [44,45].

*N*-Arylation was performed using different Buchwald co-ligands of the fourth generation [46],* i.e.*, dialkylbiaryl phosphine ligands which were optimized for different substrates. For example, RuPhos is typically used for coupling of secondary amines in high yields [47]. Therefore, the used catalyst system was confined on one group.

Typically, for Pd-mediated *N*-arylation a non-polar solvent like toluene is used together with a strong base like sodium or potassium *tert*-butoxide. Normally, weak bases are only used when excellent tolerance of functional groups is required. With toluene and potassium *tert*-butoxide high RCYs of about 70% were reached with the ligands DavePhos and RuPhos within 10–15 min reactions time. This is in agreement with the findings of Wüst* et al.* [18] who obtained similar yields by coupling an indole derivative under conditions given in Table 1 under entry 1.

The necessity of a separation and purification of [^18^F]**6** led to a considerable loss of product especially when starting with the bis(4-iodophenyl)iodonium salt (**3b**) in DMF as ^18^F-labelling precursor which produced a colloidal precipitate upon dilution with water. Furthermore, the bad miscibility of toluene and water causes problems in cartridge-based separation-processes. For an optimal performance of the reaction the same solvent should be used for both, labelling and the cross-coupling process, in order to avoid those problems and to simplify the work-up procedure. DMF is the solvent to consider in the first place for this purpose since it is typically used for ^18^F-fluorination of iodonium-precursors. However, catalyst/base systems which result in best coupling yields within short reaction time in toluene show no conversion when applied in DMF, even after extension of the reaction time to 1 h (see Table 1).

This is due to a possible side reaction of the HBC during the catalytic circle. While normally a reductive amination takes place as last step to produce the desired N-aryl compound, a β-hydride-elimination can lead to the dehalogenated product and a tetrahydropyrazine. As Christensen* et al.* [48] describe, this path becomes dominant when deprotonation is slow which normally occurs in a polar solvent like DMF, DMSO or dimethylacetamide (DMAA) under the employed conditions including Na*t*OBu as base.

Otherwise the successful use of polar solvents [49,50] and even water [51] in HBC is known. In fact, a lot of catalyst/base systems could be found which lead to high coupling yields in DMF up to 88% within 15 min which is an acceptable reaction time for multi-step ^18^F-chemistry. The pivotal point is the use of a weak base like K_3_PO_4_ or Cs_2_CO_3_. Here small but clearly higher yields are obtained with K_3_PO_4_ in DMF. In DMSO even higher yields were observed with a reverse trend concerning the use of K_3_PO_4_ and Cs_2_CO_3_ (Table 1, entry 10–19). The system Pd_2_(dba)_3_, RuPhos and Cs_2_CO_3_ in DMSO lead to a nearly complete conversion within 15 min at 100 °C. In all solvents, a double coupling of n.c.a. 4-[^18^F]fluoroiodobenzene with piperazine was not observed, which is probably due to the low concentration of the n.c.a. radiotracer [^18^F]**4** present in the reaction mixture. Another possible side reaction, the coupling of 4-[^18^F]fluorophenylpiperazine with excessive precursor, which was not separated from the reaction mixture, was also not found.

**Table 1:** HBC of 1-[^18^F]fluoro-4-iodobenzene ([^18^F]**4**) and piperazine. Total RCY is related to coupling yields based on [^18^F]**4**.

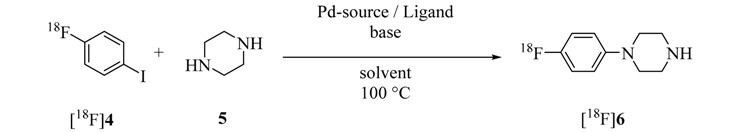				
Entry	Catalyst System	Base	Solvent	Radiochemical Conversion Yield/Reaction Time
1	Pd_2_(dba)_3_/DavePhos	NaO *t*Bu	Toluene	70%/15 min
2	Pd_2_(dba)_3_/RuPhos	NaO *t*Bu	Toluene	73%/15 min
3	[CinnamylPdCl]_2_/BrettPhos	NaO *t*Bu	Toluene	25%/30 min
4	Pd(OAc)_2_/RuPhos	NaO *t*Bu	Toluene	60%/5 min 74%/10 min
5	Pd_2_(dba)_3_/DavePhos	NaO *t*Bu	1,4-dioxane	40%/30 min
6	Pd_2_(dba)_3_/DavePhos	NaO *t*Bu	DMF	0%/60 min
7	Pd(OAc)_2_/RuPhos	NaO *t*Bu	DMF	0%/60 min
8	[P(*t*Bu)_3_PdBr]_2_	K_3_PO_4_	DMF	0%/60 min
9	[P(*t*Bu)_3_PdBr]_2_	K_2_CO_3_	DMF	0%/60 min
10	Pd_2_(dba)_3_/RuPhos	K_3_PO_4_	DMF	50%/6 min 88%/15 min
11	Pd[P(tBu)_3_]_2_	K_3_PO_4_	DMF	31%/15 min
12	Pd_2_(dba)_3_/JohnPhos	K_3_PO_4_	DMF	67%/15 min
13	Pd_2_(dba)_3_/JohnPhos	Cs_2_CO_3_	DMF	51%/15 min
14	Pd_2_(dba)_3_/XPhos	K_3_PO_4_	DMF	28%/15 min
15	Pd_2_(dba)_3_/XPhos	Cs_2_CO_3_	DMF	11%/15 min
16	Pd_2_(dba)_3_/RuPhos	K_3_PO_4_	DMSO	60%/15 min
17	Pd_2_(dba)_3_/RuPhos	Cs_2_CO_3_	DMSO	~95%/15 min
18	Pd_2_(dba)_3_/JohnPhos	K_3_PO_4_	DMSO	82%/15 min
19	Pd_2_(dba)_3_/XPhos	K_3_PO_4_	DMSO	77%/15 min

The necessity of a weaker base is surprising since a deprotonation process is estimated to be inferior under this condition. Otherwise, the strength of the base does not seem to have a considerable influence on the coupling reaction. The anion activator Kryptofix^®^2.2.2./K_2_CO_3_ used for ^18^F-fluorination is still present in the DMF solution, which is strongly and basically due to the chelated potassium. Here, deiodination is still comparatively low, but the fact that in general highest yields are obtained in DMSO can be a result of the weaker basic conditions during labelling, when tetrabutyl-ammonium bicarbonate was used as anion activator as was done with precursor **3c**. The results do not allow for a definite conclusion on the mechanism during this step of the HBC. However, the reason for slow deprotonation in polar media may be a steric hindrance of base due to coordination with the solvent. Carbonate and phosphate which can also coordinate to palladium may have better kinetics during this step due to their multidentate character.

### 2.2. Synthesis and Evaluation of the D_4_ Agonist [^18^F]FAUC 316

**Figure 3:**
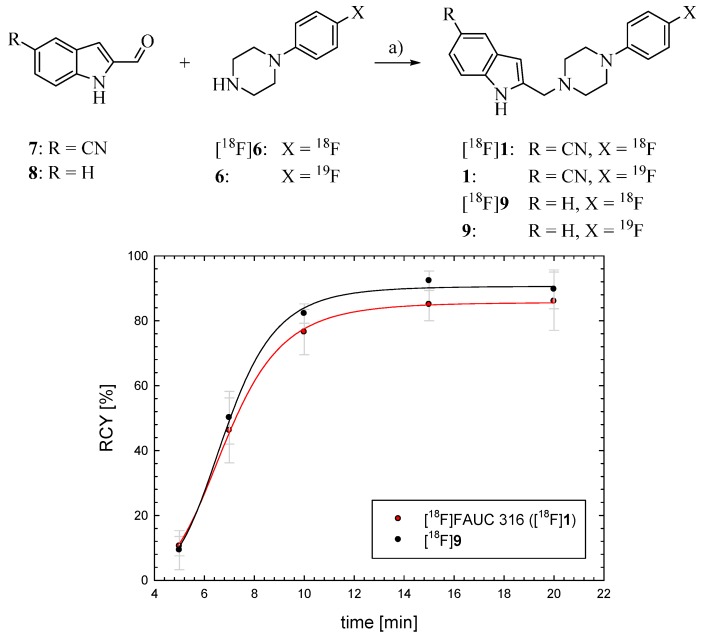
Time dependence of the reductive amination of the indole-2-carbaldehydes **7** and **8** with 1-(4-fluorophenyl)piperazine yielding [^18^F]FAUC 316 ([^18^F]**1**) and the corresponding ligand [^18^F]**9** without a nitrile substituent for comparison. Reaction conditions: NaBH_3_CN, AcOH, DMSO, 110 °C.

The synthesis of [^18^F]FAUC 316 was performed by a reductive amination of 1-(4-[^18^F]fluoro-phenyl)piperazine ([^18^F]**6**) with 2-formyl-1*H*-indole-5-carbonitrile (**7**) (see Figure 3). A pre-purification of 1-(4-[^18^F]fluorophenyl)piperazine ([^18^F]**6**) is essential to use it for application in a radiopharmaceutical synthesis following the HBC. The liquid-liquid extraction of [^18^F]**6** with hydrochloric acid (2 mol/L) allowed a selective separation of the reaction mixture containing amines as well as water soluble substances. The progress of extraction could easily be traced following the distribution of radioactivity between the two phases. After addition of sodium hydroxide until pH 9, organic compounds were fixed on a Sep-Pak C18 cartridge and eluted with DMSO as solvent of the following reaction. The liquid-liquid extraction was almost quantitative in contrast to a direct solid phase extraction on the weak cation exchanger Strata X-CW which is a faster process, but only provides a product yield of 20% ± 4%.

**Table 2:** Sequence of the two-step radiosynthesis of n.c.a. 2-((4-(4-[^18^F]fluorophenyl)-piperazin-1-yl)methyl)-1*H*-indole-5-carbonitrile ([^18^F]FAUC 316).

Structures	Time (min)	RCY (%)	Compounds and Conditions
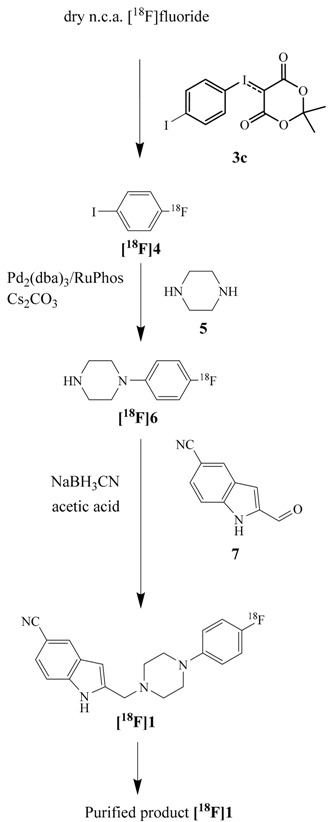	0		Drying process
	5	100	
		
		
			Addition of iodonium ylide **3c**
		
		
		
	15	70	Heating at 110 °C
		
		60	Purification step
		
			Addition of piperazine and HBC reagents
		
	30	47	Heating at 100 °C
		
		37	Purification step
		
			Addition of 2-formyl-1 *H*-indole-5-carbonitrile and NaBH_3_CN
		
		
		
		
			Stirring
	45	28	
			Pre-purification
			HPLC-separation
		
	80	10	Formulation

The synthesis of the cyanoindole precursor **7** for coupling was performed according to methods from the literature [27]. The standard compound FAUC 316 was prepared by reductive amination of commercially available 1-(4-fluorophenyl)piperazine (**2**) with the aldehyde **7** and acidic sodium cyanoborohydride in methanol obtaining a RCY of about 50%. Compound **7** was also used for radioactive coupling of the ^18^F-labelled amine ([^18^F]**6**) obtaining radiochemical yields of 85% ± 5% of [^18^F]**1** after 15 min using DMSO as solvent. In order to study whether the strong electron withdrawing effect of the cyano group of compound **7** has an influence on the reductive amination reaction, the coupling was also studied with the unsubstituted indole-2-carbaldehyde **8**. However, no significant difference in yield was observed in this case (see Figure 3). Obviously, the cyano substituent does not influence this reaction. The overall RCY of [^18^F]**1** was about 10% and 18% within 80 and 120 min, respectively, depending if a separation of the aryliodide intermediate [^18^F]**4** was necessary. Maximum molar activities of the product reached 90 GBq/µmol and the radiochemical purity was determined by HPLC to be >98%. In Table 2, the whole reaction sequence under optimized conditions is summarized.

For evaluation of the pharmacological properties of the highly affine D_4_ radioligand [^18^F]FAUC 316 for later* in vivo* application its accumulation behavior on rat brain slices was examined by* in vitro* autoradiography. Thereby, the binding of the radioligand was competed with spiperone and the inactive standard compound FAUC 316 **1**. Spiperone, an unselective but high affine dopaminergic ligand, was chosen because no applicable gold standard for D_4_ exists. The extent of competition is lower than 10% for spiperone and even less than 5% for **1**. Non-specific binding refers to the binding of a ligand to other sites in the tissue, e.g., other receptors, enzymes or membrane transporters, or to distribution of the ligand into the lipid cell membrane [52]. Therefore, in spite of the excellent affinity and selectivity reported for the inactive ligand, the preliminary* in vitro* rat brain studies with [^18^F]FAUC 316, exhibiting a comparatively high molar activity, the high non-specific binding of both radiofluorinated tracers in rat brain does not encourage to recommend them as* in vivo* PET imaging agents for dopamine D_4_ receptors in humans.

## 3. Experimental Section 

### 3.1. General

All reagents and anhydrous solvents were purchased from Aldrich (Steinheim, Germany) or Fluka (Buchs, Switzerland) and used without further purification. 2-(Hydroxylmethyl)-1*H*-indole-5-carbonitrile was available from Aurora Fine Chemicals (Graz, Austria). The preparation of 4-iodophenyltrimethylammonium triflate (**2**) [53], 4-iodophenyl-(4′-methoxyphenyl)-iodonium triflate (**3a**), bis(4-iodophenyl)iodonium triflate (**3b**) [41], (2,2-dimethyl-5,7-dioxo-1,3-dioxocan-6-yl)(4-iodophenyl)iodonium ylide (**3c**) [43], 2-formyl-1*H*-indole-5-carbonitrile [27] (**7**) and 2-((4-(4-fluorophenyl)piperazine-1-yl)methyl)-1*H*-indole [54] (**9**) were synthesized by published methods and were confirmed by NMR-spectroscopy and mass spectrometry.

Sep-Pak C-18 plus-cartridges were purchased from Waters (Eschborn, Germany), Li-Chrolut EN cartridges and glass columns (65 × 10 mm) from Merck (Darmstadt, Germany). Thin layer chromatography (TLC) was done on precoated plates of silica gel 60F_254_ (Merck) or Alumina N. The compounds were detected by UV at 254 nm. HPLC was performed on the following system from Dionex (Idstein, Germany): an Ultimate 3000 LPG-3400A HPLC pump, an Ultimate 3000 VWD-3100 UV/VIS-detector (272 nm), a UCI-50 chromatography interface, an injection valve P/N 8215. Reversed-phase HPLC was carried out using a Gemini 5 µm C18 110 Å column from Phenomenex (Aschaffenburg, Germany), with a dimension of 250 mm × 4.6 mm (flow 1 mL/min) for analytical separations and 250 mm × 10 mm (flow 5 mL/min) for semi-preparative applications. ^1^H, ^13^C and ^19^F NMR spectra were recorded on a Bruker DPX Avance 200 spectrometer with samples dissolved in CDCl_3_ or DMSO-*d*_6_. All shifts are given in δ ppm using the signals of the corresponding solvent as a reference. Mass spectra were obtained with a Finnigan Automass Multi mass spectrometer with an electron beam energy of 70 eV. High resolution electron spray mass spectra were recorded on a LTQ FT Ultra (Thermo Fischer). Melting points are uncorrected and were determined on a Mettler FP-61 apparatus in open capillaries.

### 3.2. Chemistry

*2-((4-(4-Fluorophenyl)piperazine-1-yl)methyl)-1H-indole-5-carbonitrile, FAUC 316* (**1**) (alternative to literature method [26]). 2-Formyl-1*H*-indole-5-carbonitrile (**7**) (200 mg, 1.18 mmol), 1-(4-fluorophenyl)piperazine (318 mg, 1.76 mmol), sodium cyanoborohydride (1 g, 4.72 mmol) and acetic acid (280 µL, 4.72 mmol) were dissolved in 12 mL of methanol and heated at 60 °C for 20 h. After cooling to room temperature the solution was extracted with ethyl acetate, washed with brine and dried over sodium sulfate. Upon vacuum evaporation the product was purified by flash chromatography (*n*-hexane/ethyl acetate 2:1) to obtain 190 mg (0.57 mmol, 49%) of **1**. TLC (dichloromethane/methanol 95:5): R_f_ = 0.73. ^1^H NMR (200.13 MHz, DMSO-*d*_6_) δ 2.58 (t, br, *J* = 4.16 Hz, *J* = 5.1 Hz, 4 H), 3.12 (t, br, *J* = 5.22 Hz, *J* = 4.12 Hz, 4 H), 3.73 (s, 2 H), 6.50 (s, 1 H), 6.95–7.05 (m, 4 H), 7.40 (dd, *J* = 8.42, *J* = 1.56, 1 H), 7.51 (d, *J* = 8.46, 1 H), 8.01 (s, 1 H), 11.67 (s, 1 H) ppm. ^13^C NMR (50.32 MHz, DMSO-*d*_6_) δ 49.42, 53.09, 55.25, 60.22, 101.25, 102.10, 112.73, 115.48, 115.91, 117.49, 117.64, 121.32, 123.96, 125.50, 128.14, 138.64, 139.38, 148.37, 170.78 ppm. ^19^F NMR (188.28 MHz, DMSO-*d*_6_) δ −125.61 ppm. FT-MS (ESI): 335.11 *m/z* (100) [M + H]^+^.

### 3.3. Radiochemistry

#### 3.3.1. Reactive n.c.a [^18^F]Fluoride

N.c.a. [^18^F]fluoride was produced via the ^18^O(p,n)^18^F nuclear reaction by bombardment of isotopically enriched [^18^O]water in a Ti-target [55] with 17 MeV protons at the JSW cyclotron BC 1710 (INM-5, Forschungszentrum Jülich). The produced [^18^F]fluoride was isolated from the irradiated water through electrochemical adsorption on a Sigradur-Anode (HTW Hochtemperatur-Werkstoffe GmbH, Germany) and subsequent desorption into 500 µL of pentadistilled water after recovery of the ^18^O-enriched water [56]. An aliquot of the [^18^F]fluoride solution (10–50 μL, 75–375 MBq) was filled into a 5-mL conical vial (Reactivial) containing 1 mL of acetonitrile and either 10 mg of Kryptofix^®^2.2.2 and 13 μL of an aqueous 1 M potassium carbonate solution or 80 µL of a 0.64 M tetrabutylammonium bicarbonate solution [57]. The solvent was evaporated under a stream of argon at 80 °C and 600 mbar. This azeotropic drying was repeated twice using each time 1 mL of dry acetonitrile, followed by evaporation at 8–15 mbar for 5 min.

#### 3.3.2. General Preparation of 1-[^18^F]Fluoro-4-iodobenzene ([^18^F]4)

Method A: A solution of bis(4-iodophenyl)iodonium triflate [41] (20 mg, 30 μmol) dissolved in 0.5 mL of anhydrous DMF was added to the vial containing the dried [^18^F]fluoride/Kryptofix^®^2.2.2 complex at 130 °C. Monitoring of the reaction progress was performed by radio HPLC of about 30–50 μL aliquots taken at regular time intervals (Gemini 5 μ RP18 A110, 250 × 4.6 mm, 1 mL/min, isocratic 75:25:0.5 v/v/v CH_3_CN/H_2_O/TEA) in order to determine the optimal reaction time.

After 10 min reaction time 100 mg of Celite 503 suspended in 20 mL of water was added to the reaction mixture and the total solid was removed by a LiChrolut cartridge (Merck) with a 10 µm PTFE strainer. After washing with 1 mL of water the almost clear solution was passed through a Sep-Pak C18 cartridge, which was previously conditioned with 8 mL of ethanol and 8 mL of water. After washing the cartridge with 5 mL of water and drying for 5 min by a stream of argon the product was subsequently eluted through an unconditioned Alumina N cartridge with 2 mL of anhydrous toluene into a second reaction vial.

Method B: A solution of (2,2-dimethyl-5,7-dioxo-1,3-dioxocan-6-yl)-(4-iodophenyl)iodonium ylide [43] (20 mg, 40 µmol) dissolved in 0.5 mL of anhydrous DMSO was added to the vial containing the dried [^18^F]fluoride/tetrabutylammonium bicarbonate complex at 110 °C. Monitoring of the reaction progress was done by radio HPLC of about 30–50 µL aliquots taken at regular time intervals (Gemini 5 μ RP18 A110, 250 × 4.6 mm, 1 mL/min, isocratic 75:25:0.5 v/v/v CH_3_CN/H_2_O/TEA) in order to determine the optimal reaction time. The work-up procedure is identical to that of method A.

#### 3.3.3. General Procedure of *N*-Arylation via Hartwig-Buchwald Coupling

The palladium catalyst Pd_2_(dba)_3_, palladium(π-cinnamyl) chloride dimer, or Pd(OAc)_2_ (5.5 µmol), 11 µmol of the biaryl phosphine ligand 2-dicyclohexylphosphino-2′-(*N,N*-dimethylamino)biphenyl (DavePhos), 2-dicyclohexyl-phosphino-2′,6′-diisopropoxybiphenyl (RuPhos), or 2-(dicyclohexyl­phosphino)-3,6-dimethoxy-2′,4′,6′-triisopropyl-1,1′-biphenyl (BrettPhos), 100 µmol of the base (NaOtBu or K_2_CO_3_ or K_3_PO_4_ or Cs_2_CO_3_) and 50 µmol of the amine compound were placed in a reaction vial equipped with silicon septum and a magnetic stirrer. The vial was evacuated for 30 min at 10 mbar and set under an atmosphere of dry argon Thereafter, [^18^F]**4** was directly added by passing the precursor dissolved in toluene, 1,4-dioxane or THF through an AluminaN^®^ drying cartridge into the reaction vial and the mixture was heated at 100 °C.

When DMF or DMSO was used for *N*-arylation, [^18^F]**4** was directly transferred into the second vial containing all reactants under an atmosphere of argon. Reaction progress was monitored by radio-HPLC (Gemini 5 µm RP18 A110, 250 × 4.6 mm, 1 mL/min, isocratic 75:25:0.5 v/v/v CH_3_CN/H_2_O/TEA) of aliquots of about 30–50 µL diluted tenfold with the elution solvent and typically containing about 37 kBq of activity.

#### 3.3.4. Pre-Purification of 4-[^18^F]Fluorophenylpiperazine ([^18^F]6) 

Method A: Liquid-liquid extraction of 4-[^18^F]fluorophenylpiperazine ([^18^F]**6**). The reaction mixture was extracted twice with 2 mL of hydrochloric acid (2 M) and the aqueous phases were treated with 4 mL of a 2 M sodium hydroxide solution. The solution was afterwards passed through a Sep Pak C18 cartridge, conditioned with 8 mL of ethanol and 8 mL of water, and dried for 2 min in a stream of argon. The product was then eluted with 1–2 mL of anhydrous DMSO or acetonitrile. 

Method B: Solid-liquid extraction of 4-[^18^F]fluorophenylpiperazine ([^18^F]**6**). The reaction mixture was diluted with 5 mL of methanol and passed through a Strata-X-CW weak cation exchanger cartridge, conditioned with 10 mL of methanol and 10 mL of water. After the cartridge was washed with 5 mL of an ammonium acetate buffer (pH 6.3) and 5 mL of methanol, the product was eluted with 1–2 mL of a mixture of acetonitrile and methanol 80:20 (v/v) or DMSO containing 2% of formic acid. 

#### 3.3.5. General Preparation of [^18^F]1 and [^18^F]9 by Reductive Amination

Sodium cyanoborohydride (4 mg, 64 µmol) in 50 µL of DMSO and 1*H*-indole-2-carbaldehyde (**8)** (5.8 mg, 40 µmol) or 2-formyl-1*H*-indole-5-carbonitrile (**7**) (6.8 mg, 40 µmol) in DMSO (50 µL) and acetic acid (40 µL) were added to the reaction vial containing [^18^F]**6** in DMSO. The solution was stirred for about 15 min. For determination of reaction progress the aliquots were analyzed by radio-HPLC using different systems: (A) Phenomenex Luna 5 µm C18(2) 100 Å 250 × 4 mm, 1 mL/min, isocratic 60:40:0.03 v/v/v CH_3_CN/H_2_O/TEA pH 9, (B) Phenomenex Gemini 5 µm C18 100 Å 250 × 4.6 mm, 1 mL/min, isocratic 60:40:0.03 v/v/v CH_3_CN/H_2_O/TEA pH 9, (C) Phenomenex Luna 5 µm PFP(2) 100 Å 250 × 4.6 mm, 1 mL/min, isocratic 50:50:0.01 v/v/v CH_3_CN/H_2_O/TEA pH 7.8. After addition of 20 mL of water the solution was passed through a Sep Pak C18 cartridge followed by washing with 5 mL water and drying with air. Then the cartridge was eluted with 1 mL of acetonitrile and the eluate injected on a semi-preparative HPLC system (Phenomenex Luna 5 µm PFP(2) 100 Å 250 × 10 mm, 4 mL/min, isocratic 50:50:0.01 v/v/v CH_3_CN/H_2_O/TEA pH 7.8). The collected fraction was diluted with 15 mL of water, passed through a SepPak C18 cartridge, which was then washed with 5 mL of water and dried in a stream of argon. The product was eluted from the cartridge with 4 mL of diethylether, then the solvent evaporated *in vacuo* (800 up to 330 mbar) and the product re-dissolved in 100–300 µL of ethanol. The molar activity was determined by HPLC using a UV mass calibration curve as described earlier in detail [58].

#### 3.3.6. *In Vitro* Evaluation of [^18^F]FAUC 316 ([^18^F]1)

After anesthetizing and decapitation of rats (4–6 month old female Wistar rats with 230–250 g body weight), whole brains were rapidly removed and immediately frozen at −80 °C until use. Brain sections were prepared in a cryostat microtome (CM 3050; Leica; section thickness 20 µm) at −20 °C, thaw-mounted onto silica-coated object slides, dried on silica gel overnight at 4 °C and stored at −80 °C until use. Institutional approval of animal studies was obtained from local government authorities (KFA: 9.93.2.10.35.07.244).

Incubation conditions for* in vitro* autoradiography of all tested substances were similar to those previously described by Zhang* et al.* [59]. All incubations were performed at 22 °C in Tris-HCl buffer (50 mmol/L, pH 7.4). After pre-incubation in buffer for 10 min rat brain slices were incubated in 10 nmol/L of [^18^F]FAUC 316 for 30 min either with 10 µmol/L of competitor (spiperone or standard **1**) or with the same amount of DMSO, respectively. They were washed twice for 5 min in ice-cold Tris-HCl buffer, rapidly rinsed with ice-cold distilled water, and placed under a stream of dry air for rapid drying. Object slides were exposed to γ-sensitive phosphor-imaging plates for 15–30 min and then laser scanned by a phosphor imager BAS 5000 (Fuji). The analysis of receptor autoradiography was processed according to standard image analysis software (AIDA 2.31; Raytest Isotopenmeßgeräte, Germany). Non-specific binding was defined as the residual activity in the presence of cold standard. Thus, specific binding was calculated as the difference between total and non-specific binding. 

## 4. Conclusions

Hartwig-Buchwald coupling is traditionally performed in toluene together with a strong base like sodium or potassium *tert*-butoxide. This Pd-mediated reaction was chosen in order to synthesize the intermediate n.c.a. 1-(4-[^18^F]fluorophenyl)piperazine ([^18^F]**6**) in a one-pot synthesis starting from piperazine and 1-[^18^F]fluoro-4-iodobenzene ([^18^F]**4**). Iodonium salts enabled the synthesis of 1-[^18^F]fluoro-4-iodobenzene in one step using the polar solvents DMF and DMSO with RCY up to 70%. The use of toluene and NaOtBu enables the conversion of [^18^F]**4** into [^18^F]**6** with a RCY of 74% in the presence of Pd(OAc)_2_/RuPhos. However, this necessitated the separation of [^18^F]**6** by solid phase extraction in order to transfer the intermediate into toluene as non-polar solvent which led to a considerable loss of product.

When using DMF or DMSO as solvent for the coupling, this separation step could be omitted as those solvents are typically also used for the ^18^F-labelling reaction. Thus, biarylphosphonium ligands together with the multidental inorganic bases K_3_PO_4_ or Cs_2_CO_3_ were best suited for the arylation step and led to conversion rates of more than 70% in DMF, which is comparable to those in toluene. Even in DMSO, quantitative conversation was also observed. Overall radiochemical yields of 1-(4-[^18^F]fluorophenyl)piperazine of up to 40% and 60% were reached in DMF and DMSO, respectively, depending on the labelling yield of the first step. An additional reaction step led to a quick radiosynthesis of the D_4_ agonist [^18^F]FAUC 316 in good radiochemical yields and high molar activity. The radioligand showed, however, a high non-specific binding in first* in vitro* autoradiographic studies.
